# Right and Left Ventricular Takotsubo: Same Two-Headed Monster or Different Beasts?

**DOI:** 10.7759/cureus.12225

**Published:** 2020-12-22

**Authors:** Hajir Zohourian

**Affiliations:** 1 Heart Center of Excellence, Broward Health Medical Center, Fort Lauderdale, USA

**Keywords:** takotsubo syndrome, right ventricular dysfunction, biventricular dysfunction, stress cardiomyopathy, wall motion abnormality, ventriculography

## Abstract

Right ventricular involvement in takotsubo cardiomyopathy is poorly understood and may be more common than previously reported. The significant hemodynamic ramification of this condition is elicited in this case report in which an 81-year-old female suffered a deadly biventricular takotsubo cardiomyopathy with subsequent resolution of left ventricular function but the persistence of right ventricular dysfunction. In this case, we highlight the importance of shifting focus to different recovery patterns within the two ventricles. Through a review of published cases, we have found an absence of attention to the right ventricle, an inconsistency in the appropriate diagnostic criteria of right ventricular takotsubo, and a lack of reporting on its recovery timing. This knowledge gap requires future studies.

## Introduction

Although there are few cases of isolated right ventricular takotsubo and biventricular involvement reported in publications as case reports, almost all demonstrate similar limitations in the follow-up imaging techniques to evaluate for accurate timing of recovery of the right ventricle alone or in correlation with the left ventricle. In most studies, the monitoring with a serial echocardiogram and cardiovascular magnetic resonance imaging (CMR) to confirm for recovery of ventricular function often occurred weeks after the initial diagnosis, leaving the question of exact time of recovery unanswered. The International Expert Consensus Document published in 2018 on takotsubo cardiomyopathy (TTC) expresses the concern for the presence of concomitant right ventricular involvement and how little information is known on the subject of right ventricular takotsubo [1]. This lack of information is in part due to the current design of studies, either omitting or not performing adequate imaging at shorter time intervals to investigate for right ventricular dysfunction and its recovery in correlation to the left ventricle. Thus, the timing of recovery of left versus right ventricle has not been truly investigated.

Whether the pathology of right ventricular involvement is an extension of the left ventricle or has its own unique pattern is not clear. The true diagnostic criteria for right ventricular takotsubo are not yet defined. Such criteria are essential to distinguish a true right ventricular takotsubo from either a preexisting right ventricular wall motion abnormality (WMA) in a normal subject or due to the effect of tethering from apical involvement of left ventricular takotsubo. 

This article presents a case that shows that the timing of recovery for the left and right ventricle may be different. In addition, with the review of published cases, we identify the gap in knowledge and limitations in previous investigations.

## Case presentation

An 81-year-old female with recurrent symptomatic paroxysmal atrial fibrillation who had failed prior atrial fibrillation ablations and antiarrhythmics for rhythm management was admitted for elective atrioventricular nodal ablation. Her presenting blood pressure was 140/80 mmHg, and laboratory data was stable at her baseline with hemoglobin 9 g/dL and creatinine 1.4 mg/dL. Her electrocardiogram was noted in atrial fibrillation with a rapid ventricular rate of 110 beats per minute (bpm). The atrioventricular nodal ablation and insertion of a dual-chamber permanent pacemaker were successful. Two hours after the procedure, she experienced a sudden shortness of breath and hypotension. Her new blood pressure was 88/42 mmHg, and her heart rate was 80 bpm (the heart rate was set by the pacemaker). On physical exam, jugular venous distention was noted at 9 cm H2O, and holosystolic murmur of grade 3/6 was heard at the apex.

The differential diagnosis for this sudden respiratory insufficiency and hypotension after an atrioventricular nodal ablation is extensive but includes pericardial tamponade, pulmonary embolism, stunned myocardium, retroperitoneal bleeding, and ischemia from a plaque rupture.

**Figure 1:**
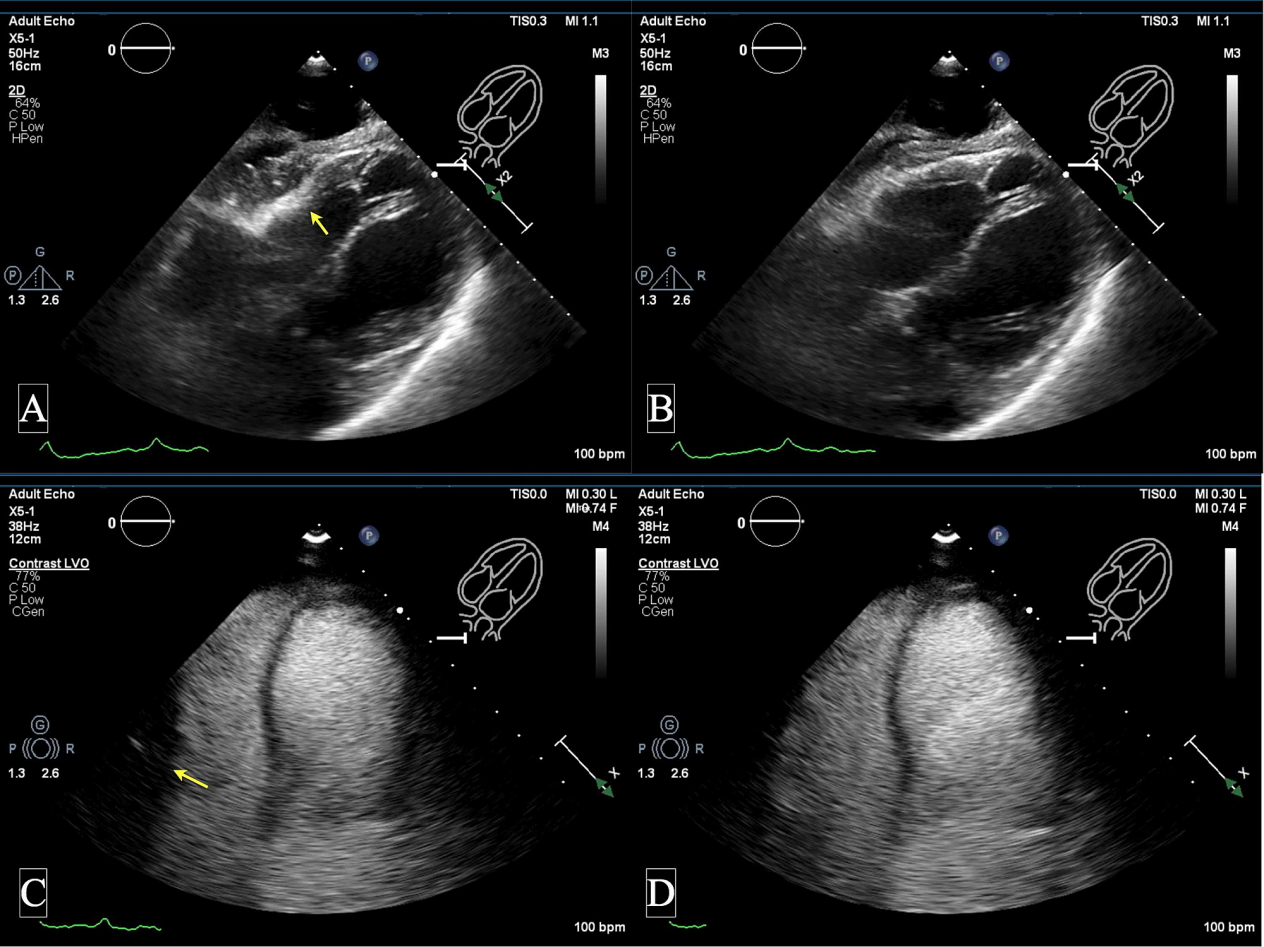
TTC: subcostal view without contrast and apical view with contrast Images are showing subcostal view without contrast (A-B); apical four-chamber view with contrast (C-D) showing biventricular takotsubo with apical akinesias and hyperdynamic basal segment (marked by the yellow arrow). TTC - takotsubo cardiomyopathy

Electrocardiogram was performed and showed new T wave inversions in inferior leads (II, III, aVF) but no ST-segment changes, which were difficult to assess due to underlying paced rhythm. Arterial blood gas showed adequate oxygenation with the partial pressure of oxygen (PaO2) of 123 mmHg on a fraction of inspired oxygen (FiO2) of 30%, placing pulmonary embolism on a lower differential diagnosis. Transthoracic echocardiogram (TTE) with contrast was performed and showed biventricular TTC (Figure 1, Video 1). The patient’s previous TTE three months ago showed normal left and right ventricular systolic function. Repeat laboratory workup revealed troponin-I of 1.45 ng/ml and brain natriuretic peptide of 1329 pg/ml.

**Video 1:** TTC: apical four-chamber view with contrast Video is showing biventricular takotsubo with apical akinesia in both LV and RV. TTC - takotsubo cardiomyopathy; LV - left ventricle; RV - right ventricle

**Figure 2:**
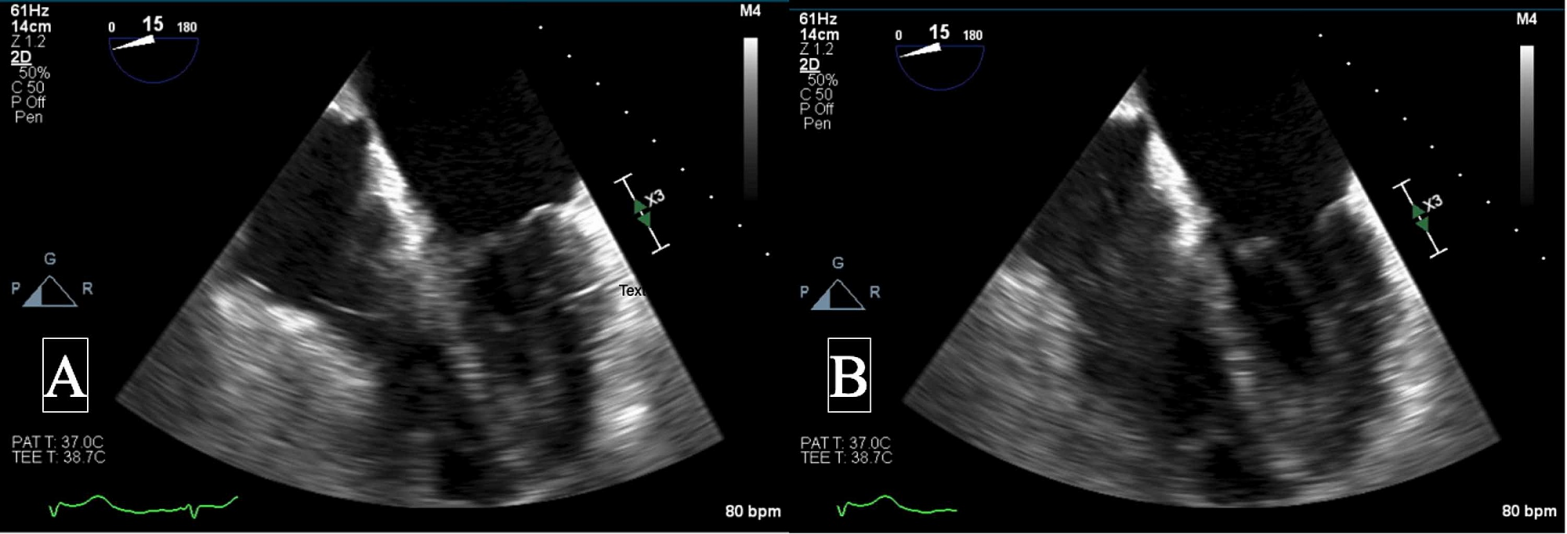
TEE: mid-esophageal four-chamber view Images are showing left ventricle (LV) systolic function recovery but persistent right ventricle (RV) apical akinesia in systolic phase (A) and diastolic phase (B). TEE - transthoracic echocardiogram

In the setting of right ventricular failure and preload dependence, 1-liter intravenous normal saline fluid recitation improved her blood pressure temporarily. Hours later, she deteriorated with a decline in mentation, poor perfusion, and blood pressure of 63/51 mmHg requiring initiation of dobutamine 10 mcg/kg/min and norepinephrine 0.2 mcg/kg/min. Multiple attempts to wean-off norepinephrine and switching dobutamine to milrinone were unsuccessful. Manual change in pacemaker setting to increase heart rate to 100 bpm did not make any significant difference in her hemodynamics. At this point, left heart catheterization was performed, confirming mild non-obstructive coronary artery disease. An intra-aortic balloon pump with 1:1 augmentation was utilized. Series of echocardiograms were performed daily. Left ventricle WMA completely resolved on day 3, while the right ventricle apical akinesia and basal hyperkinesis persisted with some improvement (Figure 2, Video 2). Repeat hemodynamic assessment in the cardiac catheterization laboratory showed left ventricle end-diastolic pressure of 14 mmHg, pulmonary artery pressure 38/22 mmHg, right ventricle (RV) pressure of 37/11 mmHg, and mean right atrial pressure of 20 mmHg. Unfortunately, with concomitant sepsis and progression of multiorgan failure, the patient expired on day 5.

**Video 2:** TEE: mid-esophageal four-chamber view and two imaging planes of LV Left ventricle (LV) systolic function has recovered, while the right ventricle (RV) apical akinesia persists.

## Discussion

This case highlights the observation of differing recovery times amongst the left ventricle and right ventricle in TTC. This observation was possible due to serial daily echocardiograms. While biventricular TTC has been reported in the literature, the recovery pattern has not been clearly elicited. Whether the same mechanisms proposed to explain left ventricular function in TTC apply to the right ventricle is unknown.

We performed an extensive electronic search in current literature and cross-referenced all published studies. After excluding studies that lacked adequate follow-up imaging modalities, we identified 14 reports that pertained to either biventricular or isolated right ventricular TTC. This review encompasses 21 total individual cases and observed “isolated” right ventricular TTC as a significant minority (Table 1).

**Table 1:** Review of published cases CE - cardiac enzyme; CMR - cardiac magnetic resonance; d - days; ECG - electrocardiogram; F - Female; LV - left ventricle; LVEF - left ventricular ejection fraction; LVG - left ventriculography; M - Male; mo - months; wk - weeks; Pts - patients; RV - right ventricle; RVG - right ventriculography; SPECT - single-photon emission computed tomography; ST↑ - ST segment elevation; ST↑↓ - ST segment elevation and depression; T↓ - T wave inversions; TTE - transthoracic echocardiogram; Apical - apical wall akinesia/hypokinesis; Basal - basal wall akinesia/hypokinesis; WMA - wall motion abnormality *  LV function improved. No mention of RV improvement. † Cardiac biopsy showed evidence for chronic myocarditis. § Follow up in eight out of nine pts. RV WMA resolved in five pts and improved in three out of eight pts. ? LV function improved from 33% to 48% after 30 days. RV dysfunction persisted.

Report	No. Pts.	Age	Sex	RV/LV	Initial LVEF	Time to improve	Time to normal	ECG	CE	RV type	LV type	Modality
Elesber AA et al. [3]	8	70 ± 13	-	Both	29% ± 9	-	86 ± 115 d	-	-	Apical	-	TTE
Haghi D et al. [4] §	9	70 ± 8	8F/1M	Both	40% ± 0.06	6-905 d	-	T↓ (5); ST↑↓ (4)	Positive	-	-	CMR
Nishikawa S et al. [6]?	1	84	F	Both	33%	14 d	30 d	ST ↑	Positive	Apical	Apical	TTE, SPECT, LVG/RVG
Nyui N, et al. [9]*	1	82	F	Both	-	8 d	35 d	ST ↑	-	Apical	Apical	TTE, LVG/RVG
Kagiyama N et al. [10]	1	89	F	RV	normal	14 d	14 d	-	Positive	Basal	Normal	CMR, TTE
Donohue D et al. [11]*	1	67	F	Both	-	7 d	7 d	T ↓	Positive	Apical	Apical	TTE
Akashi YJ et al. [12]*†	1	67	F	Both	51%	14 d	14 d	ST ↑	Positive	Apical	Apical	TTE, LVG/RVG
Novak G et al. [13]	1	65	F	Both	18%	4 d	12 d	T ↓	Positive	Apical	Apical	TTE
Bär H et al. [14]	1	80	F	Both	reduced	7 d	7 d	T ↓	Positive	Apical	Apical	TTE
Korlakunta H et al. [15]	1	89	F	Both	reduced	4 wk	4 wk	None	Positive	Apical	Apical	CMR
Akashi YJ et al. [16]	1	79	F	Both	-	3 mo	3 mo	ST ↑	-	Apical	Basal	TTE, CMR
Novak G et al. [17]	1	78	F	Both	44%	14 d	14 d	ST ↑	Positive	Apical	Apical	TTE, LVG/RVG
Citro R et al. [18]	1	81	F	RV	-	18 d	18 d	T ↓	Positive	-	Apical	TTE
Daoko J et al. [19]	1	62	F	Both	10%	9 d	5 wk	T ↓	Positive	Apical	Apical	TTE
Mrdovic I et al. [20]	1	49	F	RV	-	4 d	4 d	T ↓	Negative	Apical	-	TTE

While there are several variants of TTC, isolated left ventricular involvement has been the main focus in current literature. Unfortunately, the presence of concomitant right ventricular dysfunction was not accounted for in large systemic reviews on TTC [2]. Although right ventricular involvement has been reported sparsely, common clinical practice has yet to adjust for a more routine evaluation of right ventricular function. The prevalence of right ventricular involvement may be much higher than the previously presumed at 27% [3, 4].

The few publications that report right ventricular involvement in TTC, have significant limitations in the modality of imaging and poorly structured follow up. Limitation of imaging modality was evident in a study performed by Elesber et al. [3]. Out of 25 patients, eight patients were identified to have TCC with right ventricular involvement using visual assessment with an echocardiogram. However, out of an additional set of five patients that were assessed using CMR, four patients (80%) were noted to have right ventricular involvement. It raises the question: would the right ventricular dysfunction that was diagnosed with CMR have been diagnosed with the echocardiogram modality? Additionally, using TTC modalities such as tricuspid annular systolic excursion (TAPSE) and right ventricular fractional area change (RVFAC) have shown significant limitations due to compensating hyperdynamic wall motion in TTC and innate inaccuracy in the assessment of global right ventricular function in a single plane respectively [5].

Likewise, this limitation on follow-up was evident across several cases through our search of current literature. For example, three reports did not mention whether the right ventricular function improved at all. One report had eight out of nine patients with actual follow-up imaging. One isolated report showed that while left ventricular function improved from 33% to 48%, the right ventricular dysfunction persisted at 30 days [6]. Based on this finding, they postulated that biventricular TTC may take longer for recovery. In a report by Haghi et al., three out of eight patients with right ventricular involvement had partial improvement in function within six to 10 days, perhaps with repeat imaging done too early [4]. Similar to the above cases, our case also points toward a trend that the left and right ventricular recovery times may differ. However, there are no other cases that had follow-up imaging setup in a way to account for this discordance in recovery. 

It is crucial to note that right ventricle WMA may also coincidently exist in subjects with left ventricular TTC that may not be related to either the right or left ventricular TTC. Sievers et al. demonstrated that up to 41.4% of healthy subjects had a right ventricular WMA in a modified horizontal longitudinal plane performed with CMR [7]. Therefore, it is imperative for future studies to consider the sensitivity of CMR in the assessment of wall motion. Some of these wall motion abnormalities are pre-existing conditions that are incidentally discovered at the time of investigating for TTC. Therefore, it is necessary to have adequate follow-up imaging to ensure that the right ventricular function recovers in order to exclude unrelated diagnoses and confirm the true diagnosis of right ventricular TTC. 

Two variations of right ventricular WMA have been reported with several imaging modalities in patients with TTC. The akinetic or hypokinetic wall motion has been observed in either the apical or basal segments of the right ventricle. The basal variant is relatively rare (Table 1). The more common apical variant has a distinct right ventricular WMA that is described as hyperkinesis of the base and midsegment of the right ventricle free wall and hypokinesis of the apical wall. This feature was termed a “reverse McConnell’s sign” in a short manuscript by Liu et al. [8]. It is postulated that the hyperkinetic segment of the right ventricle free wall may simply be a physiological response to increase preload to a poor left ventricle systolic function in maintaining the cardiac output. Additionally, the hypokinesis at the apex may be due to a tethering effect from a neighboring akinetic left ventricle apex [8]. Thus, there may be a possibility that some types of right ventricle WMA may not be due to right ventricle failure but rather appear secondary to isolated left ventricle dysfunction. This observation suggests that additional studies are required to determine true diagnostic criteria for the right ventricle involvement in TTC that goes beyond visualization of WMA.

## Conclusions

The most crucial factor in diagnosing takotsubo cardiomyopathy with right ventricular involvement has a lower threshold in identifying wall motion abnormality within the right ventricle using any available imaging modality at the time of presentation. For example, the addition of a right heart ventriculogram to the usual left ventriculography should be considered and reported in future manuscripts. Study design should consider repeat imaging modalities at shorter interval periods in order to provide added information with respect to the timeline of recovery and comparison between the two ventricles. Through this case series and review of existing literature, we illustrate the gaps in knowledge and the importance of having a diagnostic criterion for right ventricular wall motion abnormality consistent with a true takotsubo cardiomyopathy. We suggest there should be an awareness of the possibility of preexisting right ventricular wall motion abnormality that is not related to takotsubo cardiomyopathy. The incidence of right ventricular involvement in takotsubo cardiomyopathy is not scarce, and its diagnosis holds important clinical implications. We recommend future studies to shift focus onto differences in left and right ventricular recovery patterns so that it can be better identified and understood.
